# A Parallel Algorithm for the Two-Dimensional Time Fractional Diffusion Equation with Implicit Difference Method

**DOI:** 10.1155/2014/219580

**Published:** 2014-03-12

**Authors:** Chunye Gong, Weimin Bao, Guojian Tang, Yuewen Jiang, Jie Liu

**Affiliations:** ^1^College of Aerospace Science and Engineering, National University of Defense Technology, Changsha 410073, China; ^2^Science and Technology on Space Physics Laboratory, Beijing 100076, China; ^3^School of Computer Science, National University of Defense Technology, Changsha 410073, China; ^4^Department of Engineering Science, University of Oxford, Oxford OX2 0ES, UK

## Abstract

It is very time consuming to solve fractional differential equations. The computational complexity of two-dimensional fractional differential equation (2D-TFDE) with iterative implicit finite difference method is *O*(*M*_*x*_ *M*_*y*_ *N* ^2^). In this paper, we present a parallel algorithm for 2D-TFDE and give an in-depth discussion about this algorithm. A task distribution model and data layout with virtual boundary are designed for this parallel algorithm. The experimental results show that the parallel algorithm compares well with the exact solution. The parallel algorithm on single Intel Xeon X5540 CPU runs 3.16–4.17 times faster than the serial algorithm on single CPU core. The parallel efficiency of 81 processes is up to 88.24% compared with 9 processes on a distributed memory cluster system. We do think that the parallel computing technology will become a very basic method for the computational intensive fractional applications in the near future.

## 1. Introduction

Building fractional mathematical models for specific phenomenon and developing numerical or analytical solutions for these fractional mathematical models are very hot in recent years. Fractional diffusion equations have been used to represent different kinds of dynamical systems [1]. But the fractional applications are rare. One reason for rare fractional applications is that the computational cost of approximating for fractional equations is too much heavy. The idea of fractional derivatives dates back to the 17th century. A fractional differential equation is a kind of equation which uses fractional derivatives. Fractional equations provide a powerful instrument for the description of memory and hereditary properties of different substances.

There has been a wide variety of numerical methods proposed for fractional equations [2, 3], for example, finite difference method [4–7], finite element method [8, 9], spectral method [10, 11], and meshless techniques [12]. Zhuang and Liu [4] presented an implicit difference approximation for two-dimensional time fractional diffusion equation (2D-TFDE) on a finite domain and discussed the stability and convergence of the method. The numerical result of an example agrees well with their theoretical analysis. Tadjeran and Meerschaert presented a numerical method, which combines the alternating directions implicit (ADI) approach with a Crank-Nicolson discretization and a Richardson extrapolation to obtain an unconditionally stable second-order accurate finite difference method, to approximate a two-dimensional fractional diffusion equation [13]. Two ADI schemes based on the *L*_1_ approximation and backward Euler method are considered for the two-dimensional fractional subdiffusion equation [14].

It is very time consuming to numerically solve fractional differential equations for high spatial dimension or big time integration. Short memory principle [15] and parallel computing [16, 17] can be used to overcome this difficulty. Parallel computing is used to solve computation intensive applications simultaneously [18–21]. Large scale applications in science and engineering such as particle transport [22–24], different linear and nonlinear systems [25], nonnumerical intelligent algorithm [26], and computational fluid dynamics [27] can rely on parallel computing. Diethelm [17] implemented the fractional version of the second-order Adams-Bashforth-Moulton method on a parallel computer and discussed the precise nature of the parallelization concept. This is the first attempt for parallel computing on fractional equations. Following that, Gong et al. [16] presented a parallel algorithm for one-dimensional Riesz space fractional diffusion equation with explicit finite difference method. The numerical solution of Riesz space fractional equations has global dependence on grid points, which means the approximation of a grid point will depend on the approximation of all grid points in one time step. The numerical solution of time fractional equations has global dependence on time steps, which means that the approximation of a grid point will depend on the approximation of the grid point in all time steps. Global dependence means the nonlocal property of fractional deviates on time or space. Explicit method is easy to be parallelized but is restrict by its stability condition. Implicit method is hard to be solved by Gauss elimination method and often uses the iterative scheme. Until today, the power of parallel computing for high dimensional and time fractional differential equations has not been tried.

This paper focuses on the two-dimensional time fractional diffusion equation studied by Zhuang and Liu [4]:

(1) ∂αu(x,y,t)∂tα=a(x,y,t)∂2u(x,y,t)∂x2 +b(x,y,t)∂2u(x,y,t)∂y2+f(x,y,t),u(x,y,0)=ϕ(x,y), (x,y)∈Ω,u(x,y,t)|∂Ω=0, t∈[0,T],

where *Ω* = {(*x*, *y*) | 0 ≤ *x* ≤ *L*_*x*_, 0 ≤ *y* ≤ *L*_*y*_, *a*(*x*, *y*, *t*) > 0, *b*(*x*, *y*, *t*) > 0}. The fractional derivative is in the Caputo form.

## 2. Background: Numerical Solution

The fractional derivative of *f*(*t*) in the Caputo sense is defined as [15]

(2) $$\begin{array}{c} \frac{\partial^{\alpha }f\left(t\right)}{\partial t^{\alpha }}=\frac{1}{\Gamma \left(1-\alpha \right)}\overset{t}{\underset{0}{\int }}\frac{f^{\prime }\left(\xi \right)}{{\left(t-\xi \right)}^{\alpha }}d\xi \;\left(0<\alpha <1\right). \end{array}$$

If *f*′(*t*) is continuous bounded derivatives in [0, *T*] for every *T* > 0, we can get

(3) ∂αf(t)∂tα=lim⁡ξ→0, nξ=t⁡ξα∑i=0n‍(−1)i(αi)=f(0)t−αΓ(1−α)+1Γ(1−α)∫0t‍  f′(ξ)(t−ξ)αdξ.

Define *τ* = *T*/*N*,*h*_*x*_ = *L*_*x*_/*M*_*x*_,*h*_*y*_ = *L*_*y*_/*M*_*y*_, *t*_*n*_ = *nτ*, *x*_*i*_ = *ih*_*x*_, and *y*_*j*_ = *jh*_*y*_, for 0 ≤ *n* ≤ *N*, 0 ≤ *i* ≤ *M*_*x*_, and 0 ≤ *j* ≤ *M*_*y*_. Let *u*_*i*,*j*_^*n*^, *φ*_*i*_^*n*^, *f*_*i*,*j*_^*n*^, *ϕ*_*i*,*j*_, *a*_*i*,*j*_^*n*^, and *b*_*i*,*j*_^*n*^ be the numerical approximation to *u*(*x*_*i*_, *y*_*j*_, *t*_*n*_), *f*(*x*_*i*_, *y*_*j*_, *t*_*n*_), *ϕ*(*x*_*i*_, *y*_*j*_), *a*(*x*_*i*_, *y*_*j*_, *t*_*n*_), and *b*(*x*_*i*_, *y*_*j*_, *t*_*n*_). We can get the implicit approximating scheme [4] for (1):

(4) ui,jn+1−ui,jn+∑s=1nbs(ui,jn+1−s−ui,jn−s) =μ1Γ(2−α)ai,jn+1(ui+1,jn+1−2ui,jn+1+ui−1,jn+1)  +μ2Γ(2−α)bi,jn+1(ui,j+1n+1−2ui,jn+1+ui,j−1n+1)  +ταΓ(2−α)fi,jn+1,

where *b*_*s*_ = (*s* + 1)^1−*α*^ − *s* ^1−*α*^  (*s* = 0,1, 2,…, *N*), *μ*_1_ = *τ* ^*α*^/*h*_*x*_^2^, and *μ*_2_ = *τ* ^*α*^/*h*_*y*_^2^. The *h*_*x*_ and *h*_*y*_ are the step size along* X* and* Y* directions defined above.

## 3. Parallel Algorithm

### 3.1. Analysis

Let *c*_1_ = *c*_1_(*i*, *j*, *k*) = *μ*_1_Γ(2 − *α*)*a*_*i*,*j*_^*n*+1^, and let *c*_2_ = *c*_2_(*i*, *j*, *k*) = *μ*_2_Γ(2 − *α*)*b*_*i*,*j*_^*n*+1^; (4) can be rewritten as

(5) −c1(ui+1,jn+1+ui−1,jn+1)+(1+2c1+2c2)ui,jn+1  −c2(ui,j+1n+1+ui,j−1n+1) =ui,jn−∑s=1nbsui,jn+1−s+∑s=1nbsui,jn−s+ταΓ(2−α)fi,jn+1.

The explicit schemes are conditionally stable and need very small *τ* for high dimensional problems for both classical and fractional equations. The implicit schemes are unconditionally stable but need to get the inverse of the coefficient matrix. Sometimes the sparse coefficient matrix is too large, making a direct method too difficult to use. So, the iterative method can be used to avoid matrix inverse:

(6) ui,jn+1,k+1 =11+2c1+2c2  ×(c1(ui+1,jn+1,k+ui−1,jn+1,k)+c2(ui,j+1n+1,k+ui,j−1n+1,k)+ui,jn    −∑s=1nbsui,jn+1−s+∑s=1nbsui,jn−s+ταΓ(2−α)fi,jn+1)

until Δ*u* = |*u*_*i*,*j*_^*n*+1,*k*+1^ − *u*_*i*,*j*_^*n*+1,*k*^| is smaller than a predefined threshold *ϵ*. *u*_0→*M*_*x*_,0→*M*_*y*__^*n*+1,*k*+1^ are the iterative variables. *u*_0→*M*_*x*_,0→*M*_*y*__^*n*^ are the known variables for the unknown *n* + 1 time step.

It is very time consuming to solve the 2D-TFDE by iterative method of (6). For determining *N*, *M*_*x*_, *M*_*y*_ and assuming if there are *K* iterations for each time step on average, there are about *M*_*x*_ *M*_*y*_(*N* ^2^/2 + 1.5*N* + 6*KN*) arithmetical logical operations ignoring the computation of the coefficients. So, the computational complexity is *O*(*M*_*x*_ *M*_*y*_ *N* ^2^), which is much more heavy than the classical integer order 2D partial differential equations *O*(*M*_*x*_ *M*_*y*_ *N*).

Besides the heavy computational cost, the memory space requirement is the other problem. Because each unknown time step needs to use all the values of the previous time steps, all the values of *u*_0→*M*_*x*_,0→*M*_*y*__^0→*N*^ need to be stored into the memory space. When *N* is big enough, the memory complexity is *O*(*M*_*x*_ *M*_*y*_ *N*), which is far bigger than the classical integer order 2D partial differential equations *O*(*M*_*x*_ *M*_*y*_).

The computation of (6) can be divided into two parts.

- Part1_*i*,*j*_ = *u*_*i*,*j*_^*n*^ − ∑_*s*=1_^*n*^ *b*_*s*_ *u*_*i*,*j*_^*n*+1−*s*^ + ∑_*s*=1_^*n*^ *b*_*s*_ *u*_*i*,*j*_^*n*−*s*^ + *τ* ^*α*^Γ(2 − *α*)*f*_*i*,*j*_^*n*+1^. The unknown value *u*_*i*,*j*_^*n*+1,*k*+1^ of grid point *P*_*i*,*j*_ at the time step *n* + 1 relies on the value of grid point *P*_*i*,*j*_ at all previous time steps of Part1_*i*,*j*_.
- Part2_*i*,*j*_ = *c*_1_(*u*_*i*+1,*j*_^*n*+1,*k*^ + *u*_*i*−1,*j*_^*n*+1,*k*^) + *c*_2_(*u*_*i*,*j*+1_^*n*+1,*k*^ + *u*_*i*,*j*−1_^*n*+1,*k*^). The unknown value *u*_*i*,*j*_^*n*+1,*k*+1^ of grid point *P*_*i*,*j*_ relies on the value of *P*_*i*+1,*j*_, *P*_*i*−1,*j*_, *P*_*i*,*j*+1_, *P*_*i*,*j*−1_.

**Figure 1:**
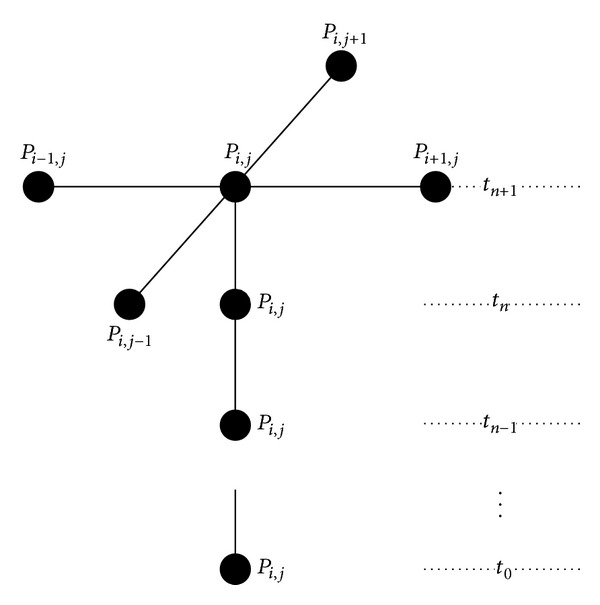
The data dependence of 2D-TFDE of grid point *P*_*i*,*j*_ of time step *t*_*n*+1_.

The data dependence of 2D-TFDE is shown in Figure 1. *u*_*i*,*j*_^*n*+1^ relies on the neighboring grid points at the same time step and the same position of all the previous time steps.

### 3.2. Task Distribution Model and Data Layout

The task distribution of the total computation should be designed on distributed memory systems, with the goal of making the total computations as efficient as possible. There are three main issues in choosing a task distribution model for these computations:

- load balance: ensure splitting of the computations reasonably evenly among all computing processors/processes throughout the time stepping;
- less communication: the task distribution model should keep the communication among different computing processes as less as possible;
- convenient programming: the parallel algorithm based on the task distribution model should not change the serial algorithm too much.

The goal of keeping attention on these issues is achieving high execution efficiency and high scalability of the parallel algorithm on distributed memory systems for 2D-TFDE.

**Figure 2:**
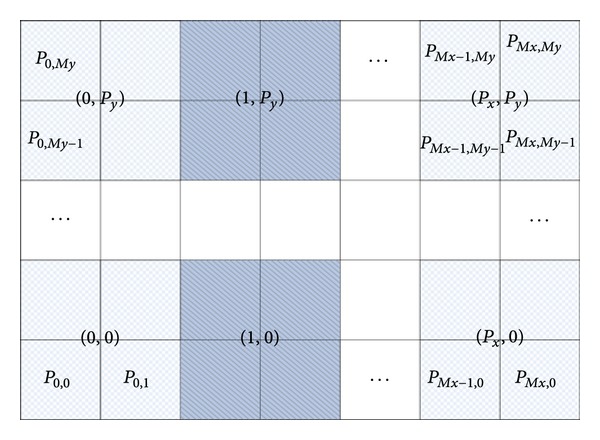
The two-dimensional task distribution model for 2D-TFDE.

Refer to (6). Part2_*i*,*j*_ computation has no data dependence. Part1_*i*,*j*_ computation has data dependence among neighboring grid points. There are mainly two kinds of task distribution models. The first one is one-dimensional distribution (ODD): splitting the domain of all grid points along the* X* or* Y* direction on average. The task distribution model of the parallel algorithm [16] for the one-dimensional Riesz space fractional equation is ODD. The parallel algorithm based on ODD will not change the serial algorithm much and the load balance is guaranteed. If task is divided along* X* direction and *M*_*y*_ is very big, the communication will influence the scalability of the parallel algorithm. The second one is two-dimensional distribution (TDD): splitting the domain of all grid points along the* X* and* Y* direction on average. So, the computing processes have a two-dimensional grid layout, with process id (*p*_*i*_, *p*_*j*_) and 0 ≤ *p*_*i*_ ≤ *P*_*x*_, 0 ≤ *p*_*j*_ ≤ *P*_*y*_. *P*_*x*_, *P*_*y*_ are the dimension size of the processes grid. The task distribution with TDD is shown in Figure 2.

**Figure 3:**
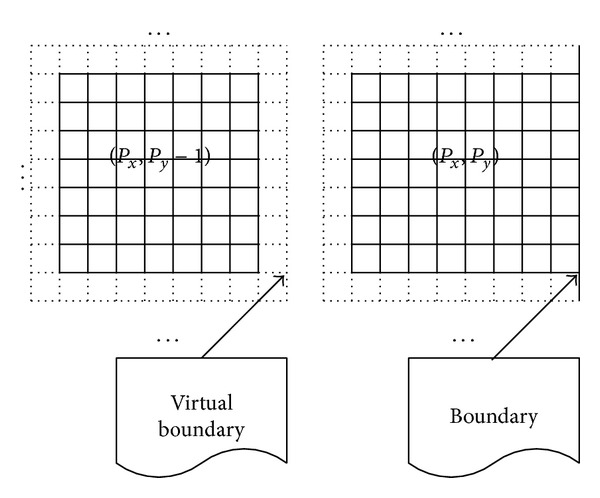
Data layout for 2D-TFDE.

With the TDD, the data layout is described in Figure 3. Each subdomain with a process may have less than four virtual boundaries to receive the boundary data from its nearest neighbors. The virtual boundary is shown with dotted lines. The process (*p*_*x*_, *P*_*y*_ − 1)(0 ≤ *p*_*x*_ ≤ *P*_*x*_) has four virtual boundaries. The process (*p*_*x*_, *P*_*y*_) only has three virtual boundaries since there is no process that stays on its right hand. A virtual boundary may have several layer grid points, which depends on the discrete scheme on space. In this paper, there is only one layer grid point for a virtual boundary with (4). In every iteration of (6), the processes exchange the data near the virtual boundaries shown in Figure 3. After the exchange, every process performs its own computation according to (6).

### 3.3. Implementation

The parallel algorithm for 2D-TFDE uses the mechanisms of process level parallelism. The process level parallelism is a kind of task level parallelism. The parallel algorithm for (1) is described in Algorithm 1.

Each process only allocates its local memory. Assuming *M*_*x*_, *M*_*y*_ are divisible by *P*_*x*_, *P*_*y*_, the process with four virtual boundaries will allocate (*M*_*x*_/*P*_*x*_ + 2)(*M*_*y*_/*P*_*y*_ + 2)*N* memory space for array *u*. The calculation of process id has three steps:

step 1: get the MPI global id ID;
step 2: *p*_*y*_ = ⌊ID/*P*_*x*_⌋;
step 3: *p*_*x*_ = ID − *P*_*x*_ *p*_*y*_.

The computations of *c*_1_(*i*, *j*), *c*_2_(*i*, *j*), *f*_*i*,*j*_, and so forth depend on the particular functions of coefficient and source terms. Performing these computations, every time step is a good choice. If these computations are performed out of the main loop (lines 9–32), a lot of memory space is required. If these computations are performed in the “While" loop (lines 16–32), it is too time consuming. The *u* ^0^ stands for the zero time step *u*_*i*,*j*_^0^ and *v* stands for *v*_*i*,*j*_. *I*_*i*,*j*_ means the iteration 1 ≤ *i* ≤ *M*_*x*_/*P*_*x*_, 1 ≤ *j* ≤ *M*_*y*_/*P*_*y*_. If a process has neighbors, it should exchange the boundary data with its neighbors. The received boundary data are stored into the designed virtual boundaries. The lines 3–7 of Algorithm 1 are the preprocessing for the parallel algorithm. The lines 9–32 are the main time marching loops. *T*_1_, *T*_2_ are used to record the execution time.

## 4. Experimental Results and Discussion

The experiment platform is a cluster with distributed memory system (DSM) architecture. One computing node consists of two Intel Xeon E5540 CPUs. The specifications of the cluster are listed in Table 1. The code runs on double precision floating point operations and is compiled by the mpif90 compiler with level three optimization (-O3). For convenience to compare the runtime, the inner loop (lines 16–32) of Algorithm 1 is fixed as 3.

### 4.1. Numerical Example and Convergence of the Parallel Algorithm

The following time fractional (*α* = 0.4) differential equation [4] was considered:

(7) ∂0.4u(x,y,t)∂t0.4=2t1.6πΓ(0.6)∂2u(x,y,t)∂x2 +t1.612πΓ(0.6)∂2u(x,y,t)∂y2+f(x,y,t),u(x,y,0)=sin(πx)sin(πy), (x,y)∈Ω,u(x,y,t)|∂Ω=0, t∈[0,T],

where *f*(*x*, *y*, *t*) = (25*t* ^1.6^/12Γ(0.6))(*t* ^2^ + 2)sin(*πx*)sin(*πy*), *Ω* = {(*x*, *y*) | 0 < *x* < 1,0 < *y* < 1}, and ∂*Ω* is the boundary of *Ω*. The exact solution of the above equation is *u*(*x*, *y*, *t*) = (*t* ^2^ + 1)sin(*πx*)sin(*πy*).

**Figure 4:**
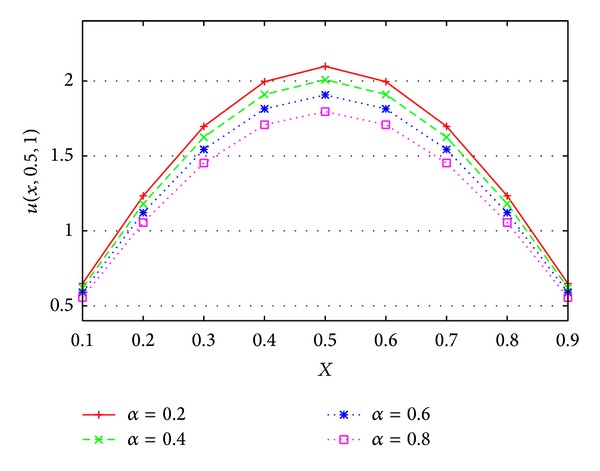
The numerical approximation whose transport is governed by the TFDE (7) for various *α* = 0.2, 0.4, 0.6, 0.8 when *y* = 0.5, *t* = 1.0.

**Figure 5:**
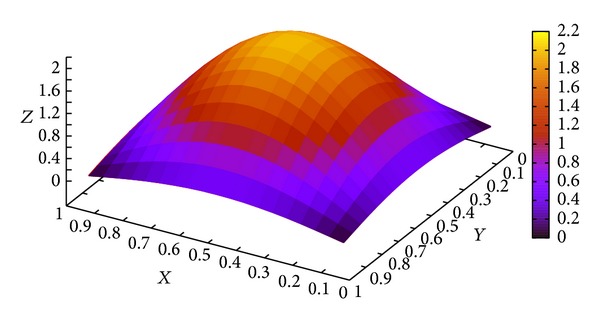
The approximation solution of (7) when *α* = 0.4 and *t* = 1.0.

The computational results for different *α* at *t* = 1.0 and *y* = 0.5 are shown in Figure 4. Figure 4 shows that the order of the fractional time derivative *α* governs the value of unknown *u*. With the increase of *α* to 1, (1) approaches the classical PDE.  Figure 5 shows the numerical solutions with *α* = 0.4, *t* = 1.0.

**Figure 6:**
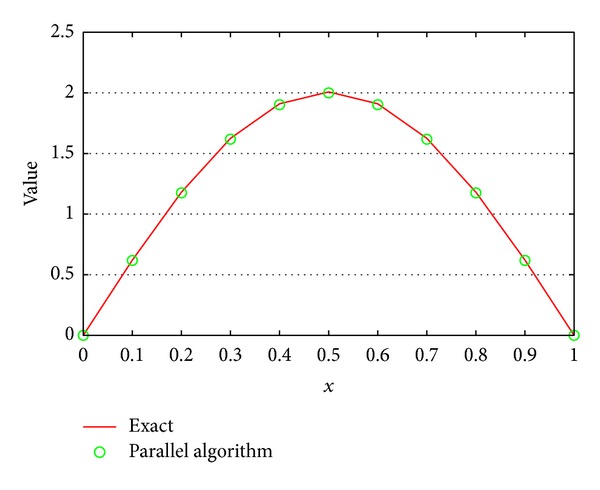
Comparison of exact solution to the solution of the parallel algorithm at time *t* = 1.0.

The parallel algorithm compares well with the exact analytic solution to the fractional partial differential equation in this test case of (7) with *α* = 0.4, shown in Figure 6. The Δ*t* and *h* are 1.0/100 and 1.0/10. The maximum absolute error is 8.36 × 10^−3^.

### 4.2. Performance Improvement

**Figure 7:**
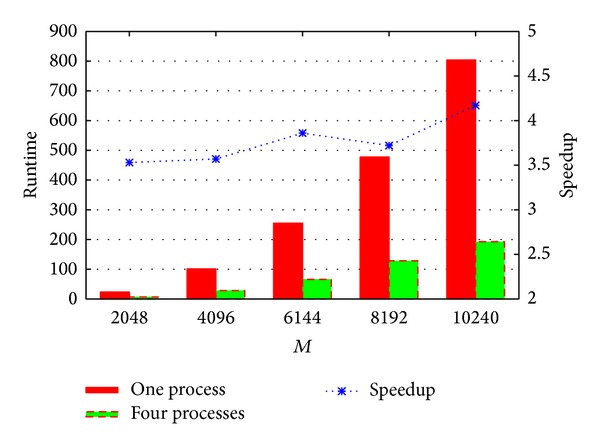
Performance comparison between one process and four processes on E5540 with fixed *N* = 10.

For fixed *N* = 10, the performance comparison between single process and four processes (single CPU) is shown in Figure 7. The* X* step number in (6) is *M*, which is the* x*-coordinate of Figure 7. *M* = *M*_*x*_ = *M*_*y*_ ranges from 2048 to 10240. With *M* = 2028, the runtime of one process is 23.45 seconds and the runtime of four processes is 6.64 seconds. The speedup is 3.53. With *M* = 10240, the runtime of one process is 803.88 seconds and the runtime of four processes is 192.76 seconds. The speedup is 4.17. From Figure 7, the parallel algorithm with fixed *N* = 10 is more than 4 times faster than the serial algorithm.

**Figure 8:**
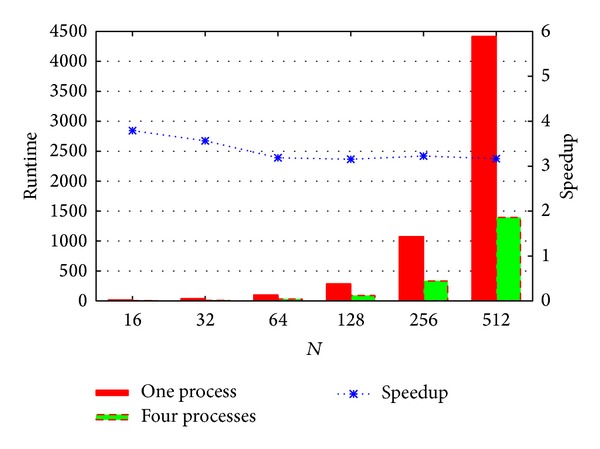
Performance comparison between one process and four processes on E5540 with fixed *M*.

For fixed *M* = 2560 = *M*_*x*_ = *M*_*y*_, the performance comparison between single process and four processes is shown in Figure 8. For single process, the* X*,* Y* step number is 2560. For four processes, the* X*,* Y* step number is 1280 with *P*_*x*_ = 2, *P*_*y*_ = 2. *N* ranges from 16 to 512. With *N* = 16, the runtime of one process is 17.63 seconds and the runtime of four processes is 4.65 seconds. The speedup is 3.79. With *N* = 512, the runtime of one process is 4415.78 seconds and the runtime of four processes is 1394.99 seconds. The speedup is 3.16. The performance of four processes is about 3.2 times higher than the performance of single process with *M* = 2560.

### 4.3. Scalability

**Figure 9:**
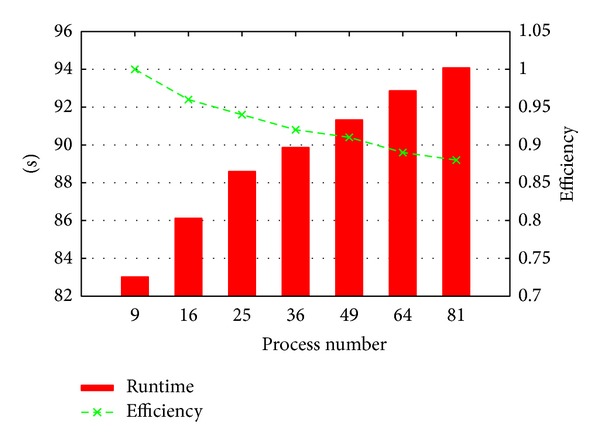
Scalability of the parallel algorithm on the cluster system.

The scalability of the parallel algorithm on the large scale cluster system is shown in Figure 9. The technical specifications of the cluster system are listed in Table 1. *N* is fixed with 10 for all conditions. Each process has the same (*M*_*x*_/*P*_*x*_, *M*_*y*_/*P*_*y*_) with *M* = *M*_*x*_ = *M*_*y*_ and *P*_*x*_ = *P*_*y*_. *M* varies from 16650, 33300, and 49950 for 9, 36, and 81 processes. The runtime of 9 processes is 83.02 seconds and the runtime of 81 processes is 94.08 seconds. The parallel efficiency of 81 processes is 88.24% compared with 9 processes. Here, the parallel efficiency is defined as the ratio of the runtime of different number of processes with the same work load on each process.

### 4.4. Discussion

The parallel Algorithm 1 will have good parallel scalability on distributed memory system. From Figure 3, we can see that each subdomain has only virtual boundary at every direction (top, bottom, left, and right). Assuming that the size of the subdomain is *M*_*a*_, *M*_*b*_(*M*_*a*_ > 0, *M*_*b*_ > 0), the inner iteration of line 16 in Algorithm 1 has about 8*M*_*a*_ *M*_*b*_ arithmetic operations with 1/(1 + 2*c*_1_ + 2*c*_2_) precomputed. It needs to establish 8 communications for neighbors except the global communication for *ϵ*. The arithmetic operation of each time step besides the inner iteration is constant as *KM*_*a*_ *M*_*b*_. *K* is bigger than 4*nM*_*a*_ *M*_*b*_. The communication data is 4*M*_*a*_ + 4*M*_*b*_ + 1 grid point. Assuming that finishing one arithmetic operation needs time *t*_*a*_ and there are *L* inner iterations, the computing time of each time step is (*K* + 8*L*)*M*_*a*_ *M*_*b*_. Assume that *t*_*b*_ is the time to establish the communication, *t*_*c*_ is the transform time for a grid point, and *t*_*d*_ is the global communication time. So, the total communication time for a time step is *L*(9*t*_*b*_ + 4*M*_*a*_ *t*_*c*_ + 4*M*_*b*_ *t*_*c*_ + *t*_*d*_). The communication/computation ratio *β* is as follows:

(8) $$\begin{array}{c} \beta =\frac{L\left(9t_{b}+4M_{a}t_{c}+4M_{b}t_{c}+t_{d}\right)}{\left(K+8L\right)M_{a}M_{b}}. \end{array}$$

The computation time is determined with the multiplication of *M*_*a*_ *M*_*b*_ and the communication time is determined with the addition of *M*_*a*_ and *M*_*b*_. The extreme of *β* is as follows:

(9) lim⁡Ma,Mb→∞L(9tb+4Matc+4Mbtc+td)(K+8L)MaMb =lim⁡Ma→∞(lim⁡Mb→∞L(9tb+4Matc+4Mbtc+td)(K+8L)MaMb) =lim⁡Ma→∞L(4tc)(K+8L)Ma=0.

That means we can enhance the parallel efficiency by enlarging the size of subdomain.

The time *t* and number of grid points will affect the convergence property. The exact solution of (7) shows that *u*(0.5,0.5, *t*) = *t* ^2^ + 1.

1. The bigger *t* becomes, the more inner iterations are needed. With *M* = *M*_*x*_ = *M*_*y*_ = 5, *N* = *M* ^2^, the first inner time step *t*_1_ needs 5 Jacobi iterations and the last inner time step *t*_*N*_ needs 31 iterations for *T* = 1.0. For *T* = 2.0, *t*_1_ becomes 7 and *t*_*N*_ becomes 61.
2. The bigger *M* becomes, the more inner iterations are needed. The *T* is fixed as 1.0. For *M* = 10, *t*_1_ becomes 6 and *t*_*N*_ becomes 66. For *M* = 10, *t*_1_ becomes 3 and *t*_*N*_ becomes 136.

The reason for the phenomenon above is that Δ*u* (*u* ^*n*+1^ − *u* ^*n*^) changes dramatically if the source term *f*(*x*, *y*, *t*) is big. The iteration times with *L* = 1.0, *M* = 15, *N* = *M* ^2^ are shown in Table 2.

The parallel algorithm is compatible with short memory principle [15]. The computing time (*K* + 8*L*)*M*_*a*_ *M*_*b*_ will become small with a smaller *K*, which is determined by *n*. The Gauss-Seidel iteration method will have better convergent speed than Jacobi iteration method, but it is hard to parallelize the Gauss-Seidel method.

As analyzed in Section 3.1, the computational complexity is *O*(*M*_*x*_ *M*_*y*_ *N* ^2^). Define the following function:

(10) $$\begin{array}{c} \begin{array}{c} w=\log_{2}\left(\sqrt{T_{2}-T_{1}}\right). \end{array} \end{array}$$

**Figure 10:**
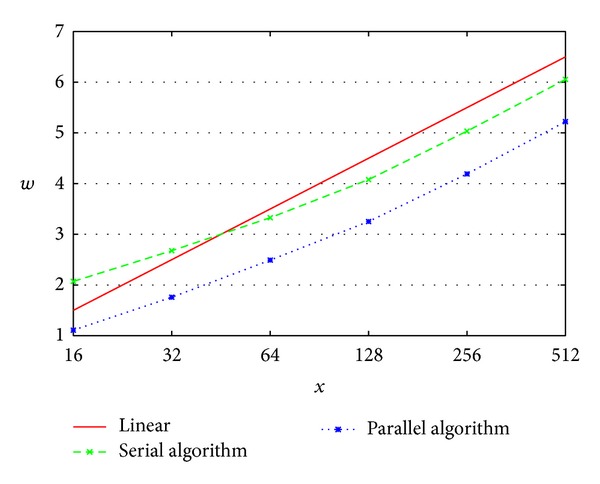
The linear variation of *w*.

*w* varies almost linearly, as shown in Figure 10. Figure 10 shows that the heavy computation is a real challenge from the point of view of computer science.

The heavy memory usage is the other challenge besides the heavy computation. Ignoring the memory usage of the coefficients and the source term *f*_*i*,*j*_^*n*^, *u*_*i*,*j*_^*n*^ needs 8*M*_*x*_ *M*_*y*_ *N* bytes memory space. It needs 100 GB memory with *M*_*x*_ = 10240,*M*_*y*_ = 10240, and *N* = 1024. As discussed above, the bigger the *M*_*x*_, *M*_*y*_ are, the smaller the *β* (communication/computation ratio) is. So, the heavy memory usage will limit the parallel efficiency of the parallel algorithm. This kind of contradictions exists in many places. One contradiction is the easy parallelization with bad convergence of the Jacobi iterative method. Another contradiction is the hard parallelization and good convergence of the Gauss-Seidel iterative method.

## 5. Conclusions and Future Work

In this paper, we present a parallel algorithm for 2D-TFDE with implicit differential method. The parallel solution is analyzed and implemented with MPI programming model. The experimental results show that the parallel algorithm compares well with the exact solution and can scale well on large scale distributed memory cluster system. So, the power of parallel computing for the time consuming fractional differential equations should be recognized.

The numerical solution for fractional equations is very computationally intensive. As a part of the future work, first, the numerical solution of high dimensional space fractional equations has global reliance on almost whole grid points, which is very challenging for real applications. Second, the Krylov subspace method with preconditioner will enhance the convergence for (4) and should be paid attention to. Third, accelerating the parallel algorithm on heterogeneous system [28] should be paid attention to.

## Figures and Tables

**Algorithm 1:**
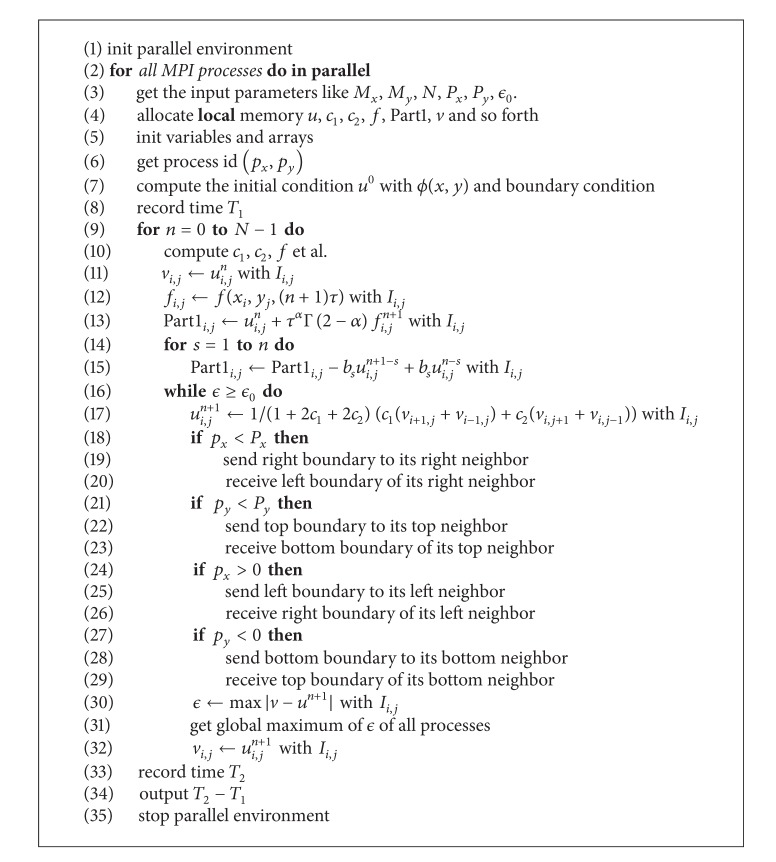
Parallel algorithm for 2D-TFDE.

**Table 1:** Technical specifications of the experiment platform.

CPU	Intel Xeon E5540, 4 cores, 2.53 GHz
Operating system	Kylin server version 3.1
Compiler	mpif90, Intel Fortran, version 11.1
Communication	MPICH2, version 1.3rc2

**Table 2:** Impact of the source term on iteration times.

*f*(*x*, *y*, *t*)	*T* = 2.0	*T* = 3.0
$\frac{25t^{1.6}}{12\Gamma (0.6)}(t^{2}+2)\sin\left(\pi x\right)\sin\left(\pi y\right)$	284	444
$\frac{25}{12\Gamma (0.6)}2\sin\left(\pi x\right)\sin\left(\pi y\right)$	253	361
$\frac{25}{12\Gamma (0.6)}\sin\left(\pi x\right)\sin\left(\pi y\right)$	245	348
$\frac{1.0}{\Gamma (0.6)}\sin\left(\pi x\right)\sin\left(\pi y\right)$	238	336
